# Risk Factor and Prediction Model for Malignant Transformation in Pancreatic Intraductal Papillary Mucinous Neoplasm

**DOI:** 10.1002/cam4.71182

**Published:** 2025-08-26

**Authors:** Dujiang Yang, Xijiao Liu, Mao Li, Zhenlu Li, Nengwen Ke, Junjie Xiong

**Affiliations:** ^1^ Division of Pancreatic Surgery, Department of General Surgery West China Hospital, Sichuan University Chengdu China; ^2^ Department of Radiology West China Hospital, Sichuan University Chengdu China

**Keywords:** intraductal papillary mucinous neoplasm, nomogram, prediction model, risk factor

## Abstract

**Purpose:**

Pancreatic intraductal papillary mucinous neoplasms (IPMN) are precursors to pancreatic cancer, with an increasing incidence due to advances in imaging techniques. This study aimed to identify risk factors for malignant transformation in IPMN and develop a predictive model using data from a large medical center in western China.

**Methods:**

Patients with IPMN admitted to West China Hospital between January 2010 and February 2022 were included in this study. They were divided into benign and malignant. Characteristic parameters and laboratory results were collected. The training set and test set were randomly divided at a ratio of 7:3. Least absolute shrinkage and selection operator regression was used to select potential prognostic factors. A nomogram was developed by logistic regression. Receiver operating characteristic curves and calibration curves were used to evaluate the model's predictive performance.

**Results:**

We retrospectively analyzed 182 patients, identifying six independent predictors of malignancy: classification, cyst wall thickening, abrupt changes in main pancreatic duct caliber, maximum tumor diameter, maximum main pancreatic duct diameter, and lnCA19‐9. We developed a nomogram with an area under the curve of 0.86 in the training set and 0.81 in the test set. The model showed strong predictive ability, providing a valuable tool for clinicians to guide preoperative decision‐making.

**Conclusion:**

Our study offers the first predictive model for malignant IPMN in western China and highlights the importance of comprehensive risk assessment, incorporating clinical, imaging, and laboratory data.

**Abbreviations**
*χ* ^2^
: chi‐square
ACM: abrupt changes in main pancreatic duct caliber
AUC: area under the curve
BD: branch duct
BMI: body mass index
CA19‐9l: carbohydrate antigen 19‐9
CEA: carcinoembryonic antigen
CP/RAP: chronic pancreatitis/recurrent acute pancreatitis
CT: computed tomography
HGD: high‐grade dysplasia
IPMN: intraductal papillary mucinous neoplasms
LASSO: least absolute shrinkage and selection operator
MD: main duct
MIX: mixed type
MLR: monocyte‐to‐lymphocyte ratio
MMPD: maximum main pancreatic duct diameter
MPD: main pancreatic duct
MRI: magnetic resonance imaging
MTD: maximum tumor diameter
NLR: neutrophil‐to‐lymphocyte ratio
PDAC: pancreatic ductal adenocarcinoma
PLR: platelet‐to‐lymphocyte ratio
ROC: receiver operating characteristic
SD: standard deviation
TCW: thickened cyst wall
WF: worrisome features

## 1 Introduction

Pancreatic intraductal papillary mucinous neoplasms (IPMN) are regarded as precursors to pancreatic cancer [1]. Recent advancements in computed tomography (CT) and magnetic resonance imaging (MRI) have significantly increased the detection of IPMN, resulting in heightened interest in their clinical management [2]. Between 1992 and 2011, the annual incidence of IPMN rose from 0.5 to 0.7 per 100,000 population [3]. IPMN are classified into three subtypes: main duct (MD), branch duct (BD), and mixed type (MIX). The incidence of pancreatic ductal adenocarcinoma (PDAC) in MD‐IPMN patients is approximately 43% (range: 11%–81%) [4], while the rate of pancreatic carcinogenesis during follow‐up for BD‐IPMN ranges from 3% to 8% [5]. The malignancy rate for MIX‐IPMN is reported to be around 33.3% [6]. Most current guidelines recommend surgical intervention for MD‐IPMN and MIX‐IPMN; however, there is considerable variation in the surgical approach for BD‐IPMN between Europe and Japan [7, 8].

The updated Fukuoka guidelines have established more stringent criteria for surgical resection, which include main pancreatic duct (MPD) dilation greater than 10 mm, jaundice, and mural nodules, among other factors [2]. These surgical criteria indicate a high likelihood of malignancy but also underscore the persistent lack of consensus regarding the definition of malignant transformation in IPMN. Consequently, identifying appropriate candidates for surgical intervention remains a significant clinical challenge. Developing an effective predictive model for malignancy could substantially benefit patients and enhance clinical decision‐making.

Predictive model is widely used in pancreatic disease [9, 10]. Previous studies have constructed predictive models for malignant IPMN based on populations in Japan [11], the United States [12, 13], and South Korea [14]. However, such studies are limited in China. Notable research conducted by Hua [15], He [16], and Huang [17] has focused on the Shanghai and Wuhan regions, respectively. Hua's study compared the performance of four existing models (Pancreatic Surgery Consortium, Japan Pancreas Society, Johns Hopkins Hospital, and Japan and Korea) in identifying malignant IPMN. However, they did not construct a predictive model [15]. He's study developed a model to predict early malignant IPMN, focusing on high‐grade dysplasia (HGD) and pT1a (invasive component ≤ 0.5 cm) [16]. In Huang et al.'s [17] study, five factors were identified, all derived from the transformation of continuous variables into binary or ternary classes—an approach that may lead to overfitting and reduced generalizability during the validation of new data.

To address these gaps, we used data from a large medical center in western China to identify risk factors for malignant transformation in pancreatic IPMN and to develop a predictive model aimed at assisting surgeons in making more precise preoperative decisions.

## 2 Methods

### 2.1 Study Design and Patient Selection

We identified consecutive patients who underwent surgery for pancreatic IPMN at West China Hospital of Sichuan University between January 2010 and February 2022 from a prospectively collected institutional database.

The inclusion criteria were as follows: (1) age over 18 years, (2) histologically confirmed pancreatic IPMN, and (3) completion of surgical intervention. The exclusion criteria included: (1) patients who underwent puncture only, (2) missing data for candidate variables, (3) non‐pancreatic tumors, and (4) indeterminate lesion locations. The study was approved by the Ethics Committee of West China Hospital and conducted in accordance with the Declaration of Helsinki.

### 2.2 Data Collection

The following clinical variables were collected: age, sex, body mass index (BMI), comorbidities (hypertension, diabetes, and chronic lung disease), smoking, drink, chronic pancreatitis/recurrent acute pancreatitis (CP/RAP), and clinical symptoms (including pancreatitis, jaundice, pain, weight loss, diarrhea, and diabetes). Tumor characteristics, including classification (MD, BD, MIX), location, tumor size, thickened cyst wall (TCW), mural nodules, enhanced mural nodules, abrupt changes in main pancreatic duct caliber (ACM), maximum tumor diameter (MTD), and maximum main pancreatic duct diameter (MMPD), were also recorded.

Laboratory data included preoperative measurements of carbohydrate antigen 19‐9 (CA19‐9), carcinoembryonic antigen (CEA), amylase, bilirubin, glucose, albumin, monocyte count, neutrophil count, lymphocyte count, platelet count, neutrophil‐to‐lymphocyte ratio (NLR), platelet‐to‐lymphocyte ratio (PLR), and monocyte‐to‐lymphocyte ratio (MLR). Imaging data, including CT and/or MRI, were reviewed by an experienced hepatopancreaticobiliary radiologist.

### 2.3 Definitions

Malignant IPMN: histologically confirmed HGD or invasive carcinoma. Benign IPMN: low‐grade and intermediate‐grade dysplasia. MPD sizes were primarily measured using magnetic resonance cholangiopancreatography or CT. Image evaluation and definitions: image assessment was independently performed by two abdominal radiologists. MD‐IPMN were defined as segmental or diffuse dilatation of the MPD > 5 mm without other causes of MPD dilation, BD‐IPMN were defined as unilocular or multilocular pancreatic cystic lesions > 5 mm that communicate with the MPD, and mixed‐type IPMN were defined as lesions meeting the diagnostic criteria for both BD and MD IPMN [8].

### 2.4 Statistical Analysis

Continuous variables are expressed as mean ± standard deviation (SD), and categorical variables are presented as frequencies and percentages. The comparison of continuous variables was made using the student's *t*‐test or Wilcoxon rank‐sum test, depending on the data distribution. Categorical variables were compared using the chi‐square (*χ* ^2^) test or Fisher's exact test. Statistical analyses were conducted using R software (Version 4.3.1).

The least absolute shrinkage and selection operator (LASSO) regression was applied to identify potential prognostic factors from the candidate variables. Logistic regression was subsequently employed to construct a nomogram. To validate the predictive model, the dataset was randomly divided into training and test sets at a 7:3 ratio. Model performance was evaluated using receiver operating characteristic (ROC) curves and calibration curves, with the area under the curve (AUC) calculated for discrimination analysis. A *p*‐value < 0.05 was considered statistically significant.

## 3 Results

### 3.1 Basic Characteristics of the Participants

**FIGURE 1:**
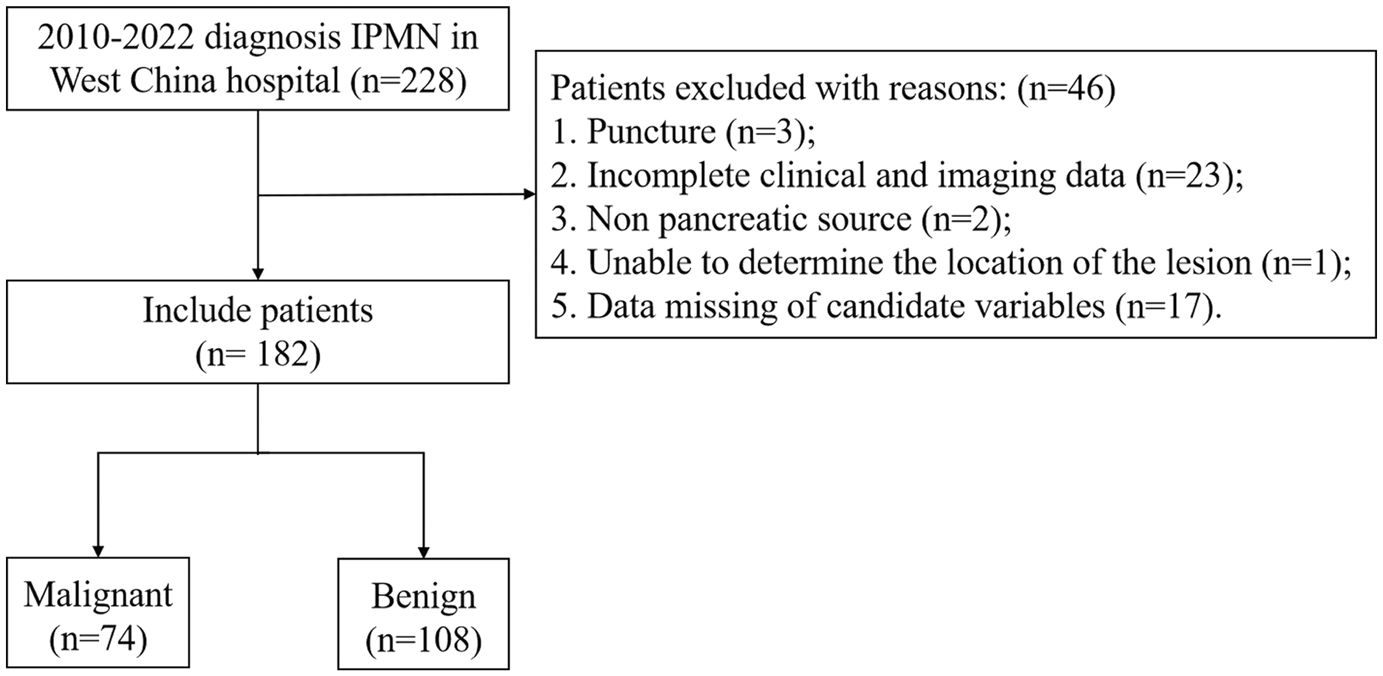
Flow chart of the study.

The flow chart of the study is presented in Figure 1. Over a 12‐year period, 228 patients with IPMN underwent surgery at West China Hospital of Sichuan University. After excluding 46 patients due to incomplete clinical or imaging data, non‐pancreatic origin, indeterminate lesion location, or missing variable data, 182 patients were included in the analysis. Among them, 74 (40.7%) were classified as malignant and 108 (59.3%) as benign. The baseline characteristics of the participants are summarized in Table 1. The mean ages in the malignant and benign groups were 62.35 ± 10.8 years and 59.36 ± 11.26 years, respectively (*p* = 0.075). Male patients accounted for 60.8% (45/74) of the malignant group and 65.7% (71/108) of the benign group. BMI was comparable between the groups (21.93 ± 3.04 vs. 21.75 ± 2.59, *p* = 0.681). The prevalence of diabetes, hypertension, and chronic lung disease was similar between the groups, as was the history of smoke and drink. Jaundice was more prevalent in the malignant group (12.2% vs. 0.9%), while pancreatitis was more common in the benign group (16.7% vs. 6.8%).

**TABLE 1:** Clinicodemographic characteristics of the overall cohort.

Variable	Benign (*N* = 108)	Malignant (*N* = 74)	*p*
Age	59.36 ± 11.26	62.35 ± 10.8	0.075
Sex (male)	71 (65.7)	45 (60.8)	0.497
BMI (kg/m^2^)	21.76 ± 2.59	21.93 ± 3.04	0.681
Comorbidity			
Diabetes mellitus	14 (13.0)	13 (17.6)	0.391
Hypertension	19 (17.6)	19 (25.7)	0.188
Chronic lung disease	5 (4.6)	3 (4.1)	1.000
Smoking	43 (39.8)	28 (37.8)	0.788
Drink	35 (32.4)	22 (29.7)	0.702
CP/RAP history	42 (38.9)	17 (23.0)	0.036
Symptoms			
Pancreatitis	18 (16.7)	5 (6.8)	
Jaundice	1 (0.9)	9 (12.2)	
Pain	72 (66.7)	45 (60.8)	
Weight loss	16 (14.8)	14 (18.9)	
Diarrhea	4 (3.7)	5 (6.8)	
Diabetes	13 (12.0)	10 (13.5)	
Other	2 (1.9)	1 (1.4)	

Abbreviations: BMI, body mass index; CP, chronic pancreatitis; *N*, number; RAP, recurrence acute pancreatitis.

Radiographic and hematological indicators characteristics are shown in Table 2. Classification (*p* < 0.001) and location (*p* = 0.019) were significantly different between the two groups. The prevalence of single tumors in the benign group (81.5%) was significantly higher than in the malignant group (64.9%) (*p* = 0.018). Thickening of cyst wall was significantly lower in the benign group (10.2%) compared to the malignant group (37.8%) (*p* < 0.001). The frequency of mural nodules was significantly lower in the benign group (75.0%) than in the malignant group (91.9%) (*p* = 0.007). Enhanced mural nodule was significantly lower in the benign group (72.2%) than in the malignant group (91.9%) (*p* = 0.002). ACM was significantly lower in the benign group (4.6%) compared to the malignant group (27.0%) (*p* < 0.001). The MTD was significantly smaller in the benign group (17.28 ± 9.02 mm) compared to the malignant group (27.61 ± 15.68 mm) (*p* < 0.001). MMPD was significantly smaller in the benign group (6.28 ± 3.67 mm) compared to the malignant group (9.89 ± 6.32 mm) (*p* < 0.001). lnCA199 in the benign group is 2.41 ± 1.12 significantly lower than the malignant group 3.21 ± 1.49, *p* < 0.001. There were no significant differences between the two groups for lnCEA (0.86 ± 0.64 vs. 1.04 ± 0.78, *p* = 0.095), glucose (6.37 ± 2.63 mmol/L vs. 7.18 ± 3.38 mmol/L, *p* = 0.069), albumin (39.42 ± 7.30 g/L vs. 38.61 ± 7.40 g/L, *p* = 0.467), monocytes (0.39 ± 0.17 vs. 0.44 ± 0.20, *p* = 0.087), neutrophils (4.72 ± 3.37 vs. 5.53 ± 4.29, *p* = 0.157), lymphocytes (1.42 ± 0.63 vs. 1.41 ± 0.56, *p* = 0.887), platelets (168.94 ± 70.55 vs. 168.86 ± 57.13, *p* = 0.994), NLR (5.34 ± 7.86 vs. 5.69 ± 8.00, *p* = 0.773), PLR (147.34 ± 115.84 vs. 140.31 ± 77.19, *p* = 0.648), and MLR (0.35 ± 0.32 vs. 0.39 ± 0.30, *p* = 0.436).

**TABLE 2:** Radiographic and hematological indicators characteristics of overall cohort.

Variable	Benign (*N* = 108)	Malignant (*N* = 74)	*p*
Classification			
Main duct	72 (66.7)	32 (43.2)	< 0.001
Branch duct	24 (22.2)	8 (10.8)	
Mixed	12 (11.1)	34 (45.9)	
Location			
Head/neck	73 (67.6)	39 (52.8)	0.019
Body/tail	20 (18.8)	12 (16.3)	
Mutilfocal	15 (13.9)	23 (31.1)	
Single tumor	88 (81.5)	48 (64.9)	0.018
TCW (mm)	11 (10.2)	28 (37.8)	< 0.001
Mural nodules (mm)	81 (75.0)	68 (91.9)	0.007
Enhanced mural nodules	78 (72.2)	68 (91.9)	0.002
ACM	5 (4.6)	20 (27.0)	< 0.001
MTD (mm)	17.28 ± 9.02	27.61 ± 15.68	< 0.001
MMPD (mm)	6.28 ± 3.67	9.89 ± 6.32	< 0.001
lnCA199	2.41 ± 1.12	3.21 ± 1.49	< 0.001
lnCEA	0.86 ± 0.64	1.04 ± 0.78	0.095
Bilirubin (μmol/L)	13.27 ± 6.82	21.40 ± 34.60	0.018
Glucose (mmol/L)	6.37 ± 2.63	7.18 ± 3.38	0.069
Albumin (g/L)	39.42 ± 7.30	38.61 ± 7.40	0.467
Monocyte (10^9^)	0.39 ± 0.17	0.44 ± 0.20	0.087
Neutrophil (10^9^)	4.72 ± 3.37	5.53 ± 4.29	0.157
Lymphocyte (10^9^)	1.42 ± 0.63	1.41 ± 0.56	0.887
Platelet	168.94 ± 70.55	168.86 ± 57.13	0.994
NLR	5.34 ± 7.86	5.69 ± 8.00	0.773
PLR	147.34 ± 115.84	140.31 ± 77.19	0.648
MLR	0.35 ± 0.32	0.39 ± 0.30	0.436

Abbreviations: ACM, abrupt changes in main pancreatic duct caliber; CA199, carbohydrate antigen 199; CEA, carcinoembryonic antigen; MLR, monocyte‐to‐lymphocyte ratio; MMPD, maximum main pancreatic duct diameter; MTD, maximum tumor diameter; *N*, number; NLR, neutrophil‐to‐lymphocyte ratio; PLR, platelet‐to‐lymphocyte ratio; TCW, thickened cyst wall.

### 3.2 Identification and Validation of Predictive Factors for IPMN With Malignant Transformation

#### 3.2.1 Variable Selection Using the LASSO Regression Model

**FIGURE 2:**
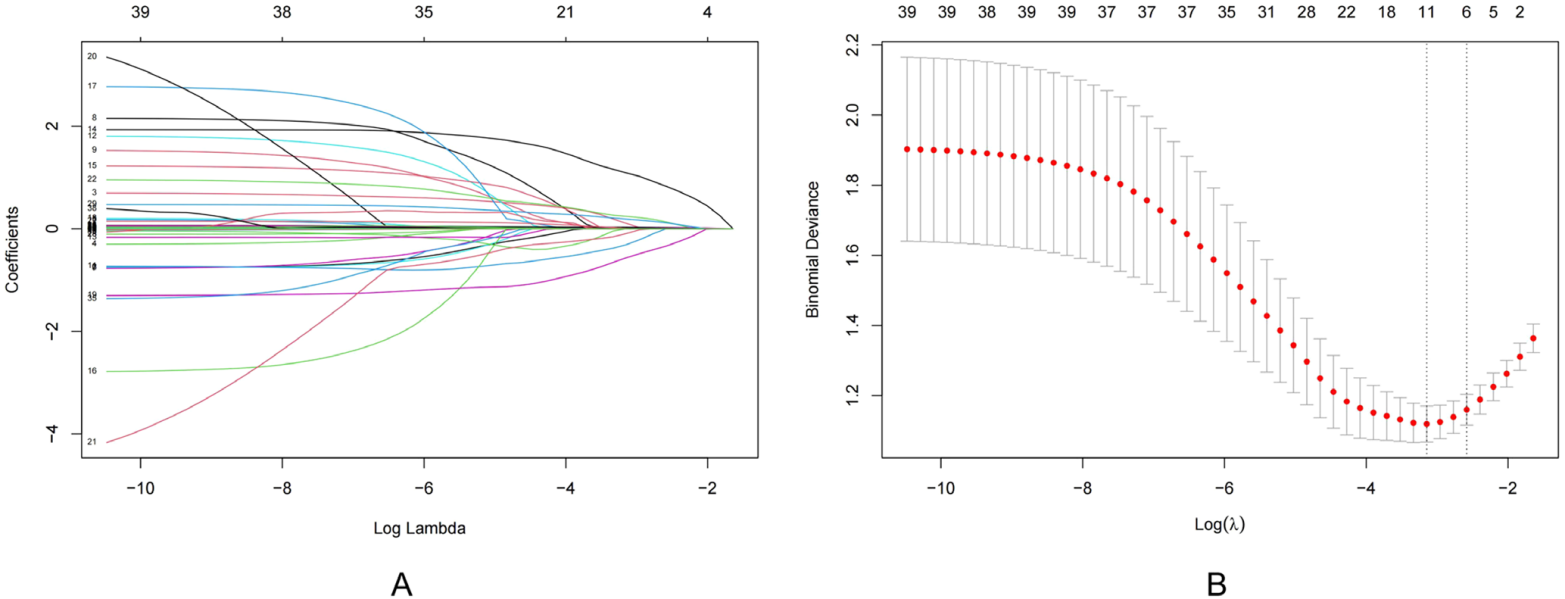
Selection of risk factors of malignant IPMN using the LASSO logistic regression algorithm. LASSO coefficient profiles of the 39 candidate variables. For the optimal lambda, six features with a non‐0 coefficient were selected.

The data were randomly divided into training and test sets at a 7:3 ratio. The characteristics of the two cohorts were presented in Table 3. Using the LASSO regression model, six variables with nonzero coefficients were identified: Classification, TCW, ACM, MTD, MMPD, and LnCA199 (Figure 2).

**TABLE 3:** Demographic and clinical characteristics of patients in training and validation set.

Variables	Training set			Validation set		
	Benign (*N* = 84)	Malignant (*N* = 44)	*p*	Benign (*N* = 24)	Malignant (*N* = 30)	*p*
Age	60.15 ± 9.67	62.18 ± 11.06	0.286	56.58 ± 15.56	62.60 ± 10.59	0.098
Sex, male	57 (67.9)	27 (61.4)	0.590	14 (58.3)	18 (60.0)	1.000
BMI (kg/m^2^)	21.69 ± 2.40	21.69 ± 3.05	0.991	21.98 ± 3.22	22.29 ± 3.04	0.726
Diabetes mellitus	11 (13.1)	9 (20.5)	0.405	3 (12.5)	4 (13.3)	1.000
Hypertension	16 (19.0)	11 (25.0)	0.578	3 (12.5)	8 (26.7)	0.345
Chronic lung disease	3 (3.6)	1 (2.3)	1.000	2 (8.3)	2 (6.7)	1.000
Smoking	35 (41.7)	15 (34.1)	0.520	8 (33.3)	13 (43.3)	0.64
Drink	27 (32.1)	14 (31.8)	1.000	8 (33.3)	8 (26.7)	0.816
CP/RAP history	35 (41.7)	10 (22.7)	0.053	7 (29.2)	7 (23.3)	0.862
Jaundice	4 (4.8)	6 (13.6)	0.153	2 (8.3)	6 (20.0)	0.416
Classification						
Main duct	57 (67.9)	19 (43.2)	< 0.001	15 (62.5)	13 (43.3)	0.021
Branch duct	18 (21.4)	5 (11.4)		6 (25.0)	3 (10.0)	
Mix	9 (10.7)	20 (45.5)		3 (12.5)	14 (46.7)	
Single tumor	68 (81.0)	25 (56.8)	0.007	20 (83.3)	23 (76.7)	0.791
TCW (mm)	9 (10.7)	17 (38.6)	< 0.001	2 (8.3)	11 (36.7)	0.036
Mural nodules (mm)	65 (77.4)	41 (93.2)	0.045	16 (66.7)	27 (90.0)	0.076
Enhanced mural nodules	63 (75.0)	41 (93.2)	0.024	15 (62.5)	27 (90.0)	0.037
ACM	79 (94.0)	34 (77.3)	0.012	0 (0.0)	10 (33.3)	0.005
MTD (mm)	17.44 ± 9.10	28.16 ± 13.58	< 0.001	16.71 ± 8.90	26.80 ± 18.55	0.018
MMPD (mm)	6.37 ± 3.87	10.34 ± 7.01	< 0.001	5.96 ± 2.88	9.23 ± 5.19	0.008
lnCA199	2.51 ± 1.13	3.35 ± 1.41	< 0.001	2.06 ± 1.03	3.01 ± 1.61	0.016
lnCEA	0.87 ± 0.66	1.09 ± 0.79	0.098	0.82 ± 0.55	0.96 ± 0.78	0.466
Bilirubin (μmol/L)	13.25 ± 6.36	18.67 ± 27.99	0.092	13.35 ± 8.41	25.40 ± 42.71	0.180
Glucose (mmol/L)	6.31 ± 2.43	7.75 ± 3.78	0.010	6.56 ± 3.28	6.35 ± 2.53	0.797
Albumin (g/L)	39.57 ± 6.95	38.28 ± 7.58	0.334	38.88 ± 8.55	39.10 ± 7.22	0.918
Monocyte (10^9^)	0.41 ± 0.17	0.43 ± 0.18	0.427	0.33 ± 0.14	0.44 ± 0.23	0.04
Neutrophil (10^9^)	4.97 ± 3.57	5.57 ± 4.45	0.404	3.86 ± 2.44	5.47 ± 4.13	0.099
Lymphocyte (10^9^)	1.41 ± 0.64	1.46 ± 0.54	0.684	1.46 ± 0.62	1.33 ± 0.59	0.467
Platelet	168.21 ± 63.85	168.57 ± 57.60	0.975	171.50 ± 91.84	169.30 ± 57.42	0.915
NLR	5.48 ± 7.70	5.72 ± 8.93	0.876	4.85 ± 8.56	5.64 ± 6.56	0.702
PLR	149.89 ± 114.87	133.53 ± 75.95	0.396	138.41 ± 121.24	150.24 ± 79.21	0.668
MLR	0.37 ± 0.35	0.37 ± 0.30	0.988	0.28 ± 0.20	0.41 ± 0.30	0.071

Abbreviations: ACM, abrupt changes in main pancreatic duct caliber; BMI, body mass index; CA199, carbohydrate antigen 199; CEA, carcinoembryonic antigen; CP, chronic pancreatitis; MLR, monocyte‐to‐lymphocyte ratio; MMPD, maximum main pancreatic duct diameter; MTD, maximum tumor diameter; *N*, number; NLR, neutrophil‐to‐lymphocyte ratio; PLR, platelet‐to‐lymphocyte ratio; RAP, recurrence acute pancreatitis; TCW, thickened cyst wall.

#### 3.2.2 Logistic Regression Development and Validation Prediction Model

**FIGURE 3:**
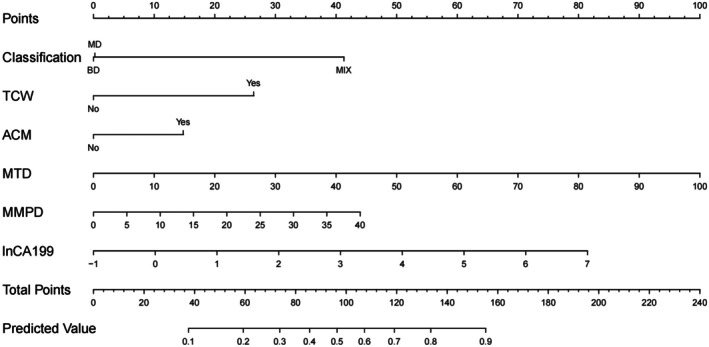
Nomogram for predicting malignant IPMN. Nomogram including four risk factors (classification, thickened cyst wall [TCW], abrupt changes in main pancreatic duct caliber [ACM], maximum tumor diameter [MTD], and maximum main pancreatic duct diameter [MMPD], and lnCA19‐9 were identified as risk factors) to predict malignant IPMN.

**FIGURE 4:**
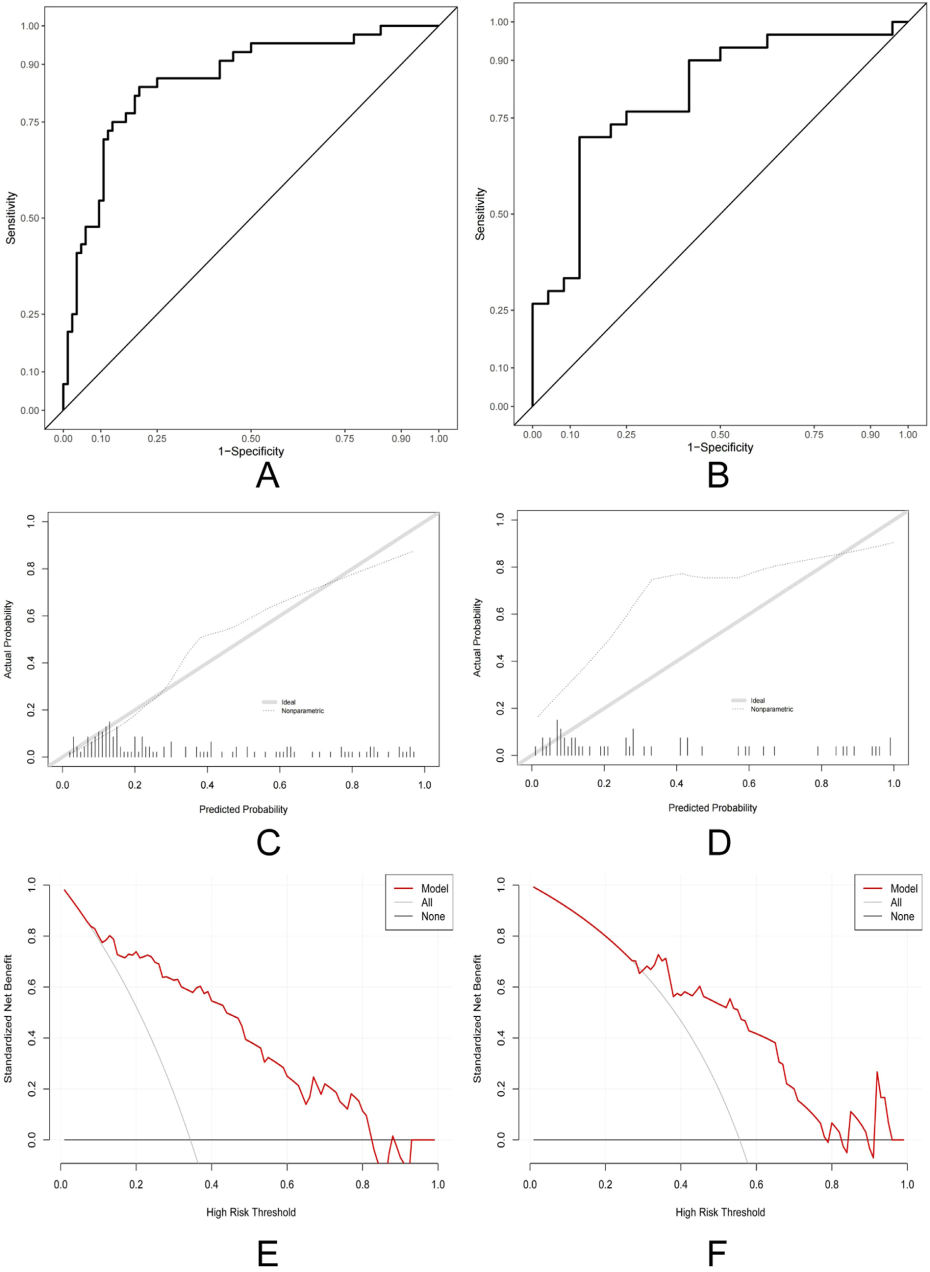
Performance of the nomogram in malignant IPMN. (A) Receiver operating characteristic curves in the training set; (B) receiver operating characteristic curves in test set; (C) calibration curves of training set; (D) calibration curves of the test set; (E) decision curve analysis of training set; (F) decision curve analysis of test set.

The six selected variables were incorporated into a nomogram model (Figure 3). ROC and calibration curves demonstrated strong predictive performance in both training and test sets, with areas under the curve (AUC) of 0.86 and 0.81, respectively (Figure 4). The sensitivity and specificity are 0.80 and 0.84 in the training set. The sensitivity and specificity are 0.88 and 0.70 in the test set. Positive and negative predictive values were 0.905 and 0.685 in the training set. Positive and negative predictive values were 0.7 and 0.875 in the test set. Calibration curves are displayed in Figure 4C,D. Brier scores of train and test are 0.142 and 0.238. Hosmer‐Lemeshow test in train and test are 5.29 (*p* = 0.73) and 34.21 (*p* < 0.001). DCA are displayed in Figure 4E,F.

## 4 Discussion

Pancreatic IPMN is a recognized precancerous lesion, with malignant transformation often necessitating surgical intervention. However, variations in surgical guidelines and physician decision‐making continue to exist. Previous studies have predominantly focused on specific subtypes of IPMN, which limits their generalizability. Our study contributes to this field by retrospectively analyzing data from a large tertiary general hospital in western China, identifying six independent predictors of malignant IPMN. We also constructed a nomogram model demonstrating robust predictive accuracy.

In this retrospective study, 182 patients (74 with malignant IPMN) were included. The adequacy of the sample size in this study is debated. On the one hand, many studies follow the widely cited rule of needing at least 10 events per variable (10 EPV). On the other hand, some studies suggest a higher EPV or the use of formulas [18, 19]. We understand that a larger sample size leads to more accurate predictive model development. Currently, the single‐center IPMN sample size in this study is relatively large. However, it still carries a risk of overfitting. We have established a prospective database and will increase the sample size in future studies to ensure sufficient sample size.

In our study, 57.1% of cases were classified as MD‐IPMN, 17.4% as BD‐IPMN, and 25% as MIX‐IPMN. Notably, MIX‐IPMN was independently associated with an increased risk of malignancy. This subtype presents unique challenges, as it combines features of both MD and BD‐IPMN. While surgical resection is frequently endorsed for MD‐IPMN, the necessity for surgery in BD‐IPMN remains a contentious issue; many studies have excluded type‐based analyses. Thus, most studies only focus on the BD‐IPMN [14, 20, 21, 22]. Type of IPMN was not explored as a risk factor in these studies. It is essential for future studies to investigate how mixed features influence clinical outcomes and recurrence rates.

Thickened cyst walls (> 2 mm), indicative of granulation tissue and fibrosis, were a key imaging marker of malignancy [23, 24, 25, 26]. Numerous studies have identified feature as one of the worrisome features (WF) [8, 27]. Similarly, ACM and larger tumor and duct diameters are well‐documented WFs [28]. Regarding the MTD and MMPD, we did not convert these continuous variables into binary variables. Previous studies have shown that a MTD greater than 3 cm is WF and a pancreatic duct diameter exceeding 10 mm is high‐risk stigmata. In our study, CA19‐9 was logarithmically transformed to account for skewed distribution, reaffirming its value as a marker for pancreatic malignancies [29]. However, elevated CA19‐9 less frequently in those with HGD. So, CA19‐9 may not be elevated until invasive cancer is present in IPMN [30].

Based on these factors, we developed a nomogram to predict the malignant IPMN. Some studies have built the predictive nomogram for malignant IPMN in single or multicenter [17, 31, 32]. In Jung's multicenter study, values for the nomogram predicting malignancy were 0.745 for Eastern, 0.856 for, Western and 0.776 for combined cohorts [32]. The latest update to the AJCC/UICC staging system categorizes typical small invasive cancers in IPMNs into three types: pT1a (≤ 0.5 cm), pT1b (> 0.5 cm, < 1 cm), and pT1c (≥ 1 cm). In He's study, only pT1a was included [16]. Thus, the predict model built by He cannot apply in all malignant IPMN. In the Huang's study, five risk factors were identified logistic screening, but all five consecutive variables were transformed into binary or ternary variables before model construction. Although the AUC of the constructed nomogram reached 0.907, the conversion of continuous variables to hierarchical variables resulted in information loss and over simplification of data, which may lead to overfitting and poor generalization ability in new data validation [17]. Fang et al. [33] proposed a noninvasive column chart based on CT features for predicting the risk of malignant IPMN, demonstrating good clinical applicability. However, it only includes the CT features and does not include important hematological indicators such as CA199. In this study, we included both imaging and hematological indicators and constructed the first predictive model for pancreatic IPMN in western China. This model has achieved good predictive, ability which can predict almost malignant patients.

Our prediction model demonstrated excellent calibration in the derivation cohort (Brier score = 0.142, Hosmer‐Lemeshow *p* = 0.73), indicating strong agreement between predicted probabilities and observed outcomes. However, test validation revealed significant calibration drift (Brier score = 0.238, Hosmer‐Lemeshow *p* < 0.001). This phenomenon is commonly observed when models are applied to populations with different risk profiles.

To evaluate potential non‐linear relationships between MTD, MMPD, lnCA19‐9, and outcome. We have conducted comprehensive analyses using restricted cubic splines. The key findings are as follows: MTD: Likelihood ratio test *p* = 0.051; MMPD: Likelihood ratio test *p* = 0.207; lnCA19‐9: Likelihood ratio test *p* = 0.162. These analyses confirm that while subtle non‐linear patterns exist, they do not significantly impact model performance or calibration in our cohort.

This study has some limitations. First, its retrospective design introduces selection bias, and being single‐center with a relatively small sample size, the findings may not be widely generalizable. Second, subtype stratification was not performed, which could affect predictive precision. Third, forty‐six patients were excluded from the final analysis due to incomplete data on key variables required for model development and validation. While this exclusion was necessary to ensure the integrity of the model‐building process using complete cases, it introduces a potential source of selection bias. Patients with incomplete data might systematically differ from those with complete data in terms of demographics, disease characteristics, or outcomes. Finally, our ductal measurement protocol focused on maximum axial diameter, which may inadequately characterize segmental MD‐IPMN with HGD. Future studies should incorporate duct length, contour analysis, and radiomics features to better quantify malignant potential. Future multicenter prospective studies should address these limitations and explore the integration of molecular, imaging, and clinical data to improve predictive accuracy.

## 5 Conclusion

We identified six key risk factors for malignant IPMN and developed a robust predictive model with high accuracy. This nomogram provides a practical tool for clinical decision‐making and highlights the importance of multifactorial risk assessment in managing IPMN patients.

## Author Contributions

**Dujiang Yang:** writing – original draft, funding acquisition, investigation, methodology, data curation, validation, formal analysis. **Xijiao Liu:** methodology, investigation, writing – original draft, validation, data curation, formal analysis. **Mao Li:** data curation, formal analysis. **Zhenlu Li:** data curation, formal analysis. **Nengwen Ke:** writing – review and editing, conceptualization, project administration. **Junjie Xiong:** writing – review and editing, conceptualization, project administration, funding acquisition.

## Ethics Statement

This study was conducted according to the principles in the Declaration of Helsinki and was approved by the Ethics Committee of the West China Hospital. The need for consent in this study is waived by our review board.

## Conflicts of Interest

The authors declare no conflicts of interest.

## Data Availability

The data that support the findings of this study are available from the corresponding author upon reasonable request.

## References

[1] G. A. Margonis , A. Pulvirenti , V. Morales‐Oyarvide , et al., “Performance of the 7th and 8th Editions of the American Joint Committee on Cancer Staging System in Patients With Intraductal Papillary Mucinous Neoplasm‐Associated PDAC: A Multi‐Institutional Analysis,” Annals of Surgery 277, no. 4 (2023): 681–688. 34793353 10.1097/SLA.0000000000005313

[2] M. Tanaka , C. Fernández‐Del Castillo , T. Kamisawa , et al., “Revisions of International Consensus Fukuoka Guidelines for the Management of IPMN of the Pancreas,” Pancreatology 17, no. 5 (2017): 738–753. 28735806 10.1016/j.pan.2017.07.007

[3] F. H. Lui , R. Shaw , and L. B. Gerson , “Increasing Incidence of Malignant IPMN (m‐IPMN) and Poorer Survival Among African Americans: 2017 Category Award (Biliary/Pancreas): 2,” American Journal of Gastroenterology 112 (2017): S1.28981040

[4] B. L. Ecker , S. M. Dickinson , L. V. Saadat , et al., “Segmental Versus Diffuse Main Duct Intraductal Papillary Mucinous Neoplasm: Examination of Main Pancreatic Duct Morphology and Implications for Malignancy Risk and Extent of Surgical Resection,” Annals of Surgery 278, no. 1 (2023): 110–117. 35950775 10.1097/SLA.0000000000005672 PMC9918598

[5] H. Oyama , M. Tada , K. Takagi , et al., “Long‐Term Risk of Malignancy in Branch‐Duct Intraductal Papillary Mucinous Neoplasms,” Gastroenterology 158, no. 1 (2020): 226–237.e225. 31473224 10.1053/j.gastro.2019.08.032

[6] Y. Vaalavuo , M. Vornanen , R. Ahola , et al., “Long‐Term (10‐Year) Outcomes and Prognostic Factors in Resected Intraductal Papillary Mucinous Neoplasm Tumors in Finland: A Nationwide Retrospective Study,” Surgery 174, no. 1 (2023): 75–82. 37062604 10.1016/j.surg.2023.02.006

[7] European Study Group on Cystic Tumours of the Pancreas , “European Evidence‐Based Guidelines on Pancreatic Cystic Neoplasms,” Gut 67, no. 5 (2018): 789–804. 29574408 10.1136/gutjnl-2018-316027 PMC5890653

[8] T. Ohtsuka , C. Fernandez‐Del Castillo , T. Furukawa , et al., “International Evidence‐Based Kyoto Guidelines for the Management of Intraductal Papillary Mucinous Neoplasm of the Pancreas,” Pancreatology 24, no. 2 (2024): 255–270. 38182527 10.1016/j.pan.2023.12.009

[9] S. Chen , S. Ren , K. Guo , M. J. Daniels , Z. Wang , and R. Chen , “Preoperative Differentiation of Serous Cystic Neoplasms From Mucin‐Producing Pancreatic Cystic Neoplasms Using a CT‐Based Radiomics Nomogram,” Abdominal Radiology 46, no. 6 (2021): 2637–2646. 33558952 10.1007/s00261-021-02954-8

[10] S. Ren , L. Qian , M. J. Daniels , S. Duan , R. Chen , and Z. Wang , “Evaluation of Contrast‐Enhanced Computed Tomography for the Differential Diagnosis of Hypovascular Pancreatic Neuroendocrine Tumors From Chronic Mass‐Forming Pancreatitis,” European Journal of Radiology 133 (2020): 109360. 33126171 10.1016/j.ejrad.2020.109360

[11] Y. Shimizu , S. Hijioka , S. Hirono , et al., “New Model for Predicting Malignancy in Patients With Intraductal Papillary Mucinous Neoplasm,” Annals of Surgery 272, no. 1 (2020): 155–162. 30499803 10.1097/SLA.0000000000003108

[12] G. Gemenetzis , F. Bagante , J. F. Griffin , et al., “Neutrophil‐To‐Lymphocyte Ratio Is a Predictive Marker for Invasive Malignancy in Intraductal Papillary Mucinous Neoplasms of the Pancreas,” Annals of Surgery 266, no. 2 (2017): 339–345. 27631774 10.1097/SLA.0000000000001988

[13] M. A. Attiyeh , C. Fernández‐Del Castillo , M. Al Efishat , et al., “Development and Validation of a Multi‐Institutional Preoperative Nomogram for Predicting Grade of Dysplasia in Intraductal Papillary Mucinous Neoplasms (IPMNs) of the Pancreas: A Report From the Pancreatic Surgery Consortium,” Annals of Surgery 267, no. 1 (2018): 157–163. 28079542 10.1097/SLA.0000000000002015 PMC5565720

[14] J. Y. Jang , T. Park , S. Lee , et al., “Proposed Nomogram Predicting the Individual Risk of Malignancy in the Patients With Branch Duct Type Intraductal Papillary Mucinous Neoplasms of the Pancreas,” Annals of Surgery 266, no. 6 (2017): 1062–1068. 27607098 10.1097/SLA.0000000000001985

[15] J. Hua , B. Zhang , X. J. Yang , et al., “Validation and Head‐To‐Head Comparison of Four Models for Predicting Malignancy of Intraductal Papillary Mucinous Neoplasm of the Pancreas: A Study Based on Endoscopic Ultrasound Findings,” World Journal of Gastrointestinal Oncology 11, no. 11 (2019): 1043–1053. 31798784 10.4251/wjgo.v11.i11.1043 PMC6883176

[16] X. He , R. Fan , J. Sun , et al., “A Model for Predicting Degree of Malignancy in Patients With Intraductal Papillary Mucinous Neoplasm,” Frontiers in Oncology 13 (2023): 1087852. 36761937 10.3389/fonc.2023.1087852 PMC9902908

[17] X. Huang , T. Guo , Z. Zhang , et al., “Prediction of Malignant Intraductal Papillary Mucinous Neoplasm: A Nomogram Based on Clinical Information and Radiological Outcomes,” Cancer Medicine 12, no. 16 (2023): 16958–16971. 37434479 10.1002/cam4.6326 PMC10501290

[18] R. D. Riley , J. Ensor , K. I. E. Snell , et al., “Calculating the Sample Size Required for Developing a Clinical Prediction Model,” BMJ 368 (2020): m441. 32188600 10.1136/bmj.m441

[19] E. O. Ogundimu , D. G. Altman , and G. S. Collins , “Adequate Sample Size for Developing Prediction Models Is Not Simply Related to Events Per Variable,” Journal of Clinical Epidemiology 76 (2016): 175–182. 26964707 10.1016/j.jclinepi.2016.02.031 PMC5045274

[20] F. Flammia , T. Innocenti , A. Galluzzo , et al., “Branch Duct‐Intraductal Papillary Mucinous Neoplasms (BD‐IPMNs): An MRI‐Based Radiomic Model to Determine the Malignant Degeneration Potential,” La Radiologia Medica 128, no. 4 (2023): 383–392. 36826452 10.1007/s11547-023-01609-6

[21] D. Tamburrino , N. de Pretis , E. Pérez‐Cuadrado‐Robles , et al., “Identification of Patients With Branch‐Duct Intraductal Papillary Mucinous Neoplasm and Very Low Risk of Cancer: Multicentre Study,” British Journal of Surgery 109, no. 7 (2022): 617–622. 35511697 10.1093/bjs/znac103 PMC10364743

[22] R. M. Pozzi Mucelli , C. F. Moro , M. Del Chiaro , et al., “Branch‐Duct Intraductal Papillary Mucinous Neoplasm (IPMN): Are Cyst Volumetry and Other Novel Imaging Features Able to Improve Malignancy Prediction Compared to Well‐Established Resection Criteria?,” European Radiology 32, no. 8 (2022): 5144–5155. 35275259 10.1007/s00330-022-08650-5 PMC9279268

[23] T. Yamazaki , T. Tomoda , H. Kato , et al., “Risk Factors for the Development of High‐Risk Stigmata in Branch‐Duct Intraductal Papillary Mucinous Neoplasms,” Internal Medicine 60, no. 20 (2021): 3205–3211. 33967138 10.2169/internalmedicine.7168-21 PMC8580771

[24] C. E. Dewhurst and K. J. Mortele , “Cystic Tumors of the Pancreas: Imaging and Management,” Radiologic Clinics of North America 50, no. 3 (2012): 467–486. 22560692 10.1016/j.rcl.2012.03.001

[25] M. D'Onofrio , F. Vecchiato , A. Gallotti , M. Falconi , P. Capelli , and M. R. Pozzi , “Small Undifferentiated Pancreatic Adenocarcinoma Which Mimics IPMN at Imaging,” Journal of the Pancreas: JOP 10, no. 4 (2009): 406–408. 19581744

[26] B. Taouli , V. Vilgrain , D. O'Toole , M. P. Vullierme , B. Terris , and Y. Menu , “Intraductal Papillary Mucinous Tumors of the Pancreas: Features With Multimodality Imaging,” Journal of Computer Assisted Tomography 26, no. 2 (2002): 223–231. 11884778 10.1097/00004728-200203000-00011

[27] J. S. Kang , T. Park , Y. Han , et al., “Clinical Validation of the 2017 International Consensus Guidelines on Intraductal Papillary Mucinous Neoplasm of the Pancreas,” Annals of Surgical Treatment and Research 97, no. 2 (2019): 58–64. 31388508 10.4174/astr.2019.97.2.58 PMC6669133

[28] W. Kwon , Y. Han , Y. Byun , et al., “Predictive Features of Malignancy in Branch Duct Type Intraductal Papillary Mucinous Neoplasm of the Pancreas: A Meta‐Analysis,” Cancers (Basel) 12, no. 9 (2020): 2618. 32937809 10.3390/cancers12092618 PMC7563991

[29] G. Luo , K. Jin , S. Deng , et al., “Roles of CA19‐9 in Pancreatic Cancer: Biomarker, Predictor and Promoter,” Biochimica Et Biophysica Acta. Reviews on Cancer 1875, no. 2 (2021): 188409. 32827580 10.1016/j.bbcan.2020.188409

[30] K. E. Shockley , B. To , W. Chen , G. Lozanski , Z. Cruz‐Monserrate , and S. G. Krishna , “The Role of Genetic, Metabolic, Inflammatory, and Immunologic Mediators in the Progression of Intraductal Papillary Mucinous Neoplasms to Pancreatic Adenocarcinoma,” Cancers 15, no. 6 (2023): 1722. 36980608 10.3390/cancers15061722 PMC10046238

[31] S. J. Yoon , H. Kim , O. Lee , et al., “Development and External Validation of a Nomogram With Inflammatory Markers for Predicting Invasiveness of Intraductal Papillary Mucinous Neoplasm of Pancreas,” Medicine (Baltimore) 101, no. 11 (2022): e29036. 35356913 10.1097/MD.0000000000029036 PMC10684245

[32] W. Jung , T. Park , Y. Kim , et al., “Validation of a Nomogram to Predict the Risk of Cancer in Patients With Intraductal Papillary Mucinous Neoplasm and Main Duct Dilatation of 10 Mm or Less,” British Journal of Surgery 106, no. 13 (2019): 1829–1836. 31441048 10.1002/bjs.11293 PMC7875493

[33] X. Fang , F. Liu , J. Li , et al., “Computed Tomography Nomogram to Predict a High‐Risk Intraductal Papillary Mucinous Neoplasm of the Pancreas,” Abdominal Radiology 46, no. 11 (2021): 5218–5228. 34409514 10.1007/s00261-021-03247-w

